# Comparative physiological and soil microbial community structural analysis revealed that selenium alleviates cadmium stress in *Perilla frutescens*

**DOI:** 10.3389/fpls.2022.1022935

**Published:** 2022-10-07

**Authors:** Xiaohuan Yang, Ying Li, Jinhu Ma, Fei Wu, Liyin Wang, Liangliang Sun, Ping Zhang, Wenying Wang, Jin Xu

**Affiliations:** ^1^ College of Horticulture, Shanxi Agricultural University, Taigu, China; ^2^ College of Agriculture, Shanxi Agricultural University, Taigu, China; ^3^ College of Life Science, Qinghai Normal University, Xining, China

**Keywords:** selenium, cadmium stress, photosynthesis, cell wall components, soil microbial community, *Perilla frutescens*

## Abstract

Cadmium (Cd) toxicity not only affects plant growth and development, but also affects human health through the food chain. Several studies have demonstrated that Selenium (Se) alleviates Cd stress in plants; however, whether and how Se-alleviated Cd stress by regulating the structure of soil microbial community remain largely unclear. Here, we investigated the alleviating effects of exogenous applied Se (foliar spraying or root application) on plant growth under Cd stress in perilla (*Perilla frutescens* L.) by measuring the biomass, photosynthetic fluorescence parameters, root cell wall components and soil microbial community structure and diversity. Under Cd stress, perilla seedlings supplemented with Se increased chlorophyll content. Foliar spraying Se increased the levels of relative chlorophyll content (ΦII), photosynthetic system II (Φ_PSII_) and electron transport rate (ETR) in perilla leaves under Cd stress; while, root application of Se increased the levels of photosynthetic rate (Pn), stomatal conductance (Gs), transpiration rate (Tr), water use efficiency (WUE) and stomatal limitation value (Ls) under Cd stress. Compared with Cd toxicity alone, root application of Se increased the contents of hemicellulosic 1 and hemicellulosic 2 in the cell wall of perilla roots. Cd toxicity or root application of Se did not affect soil bacterial community diversity. Root application of Se increased the relative abundance of *Proteobacteria*, *Bacteroidetes*, *Fibrobacteres*, *Sphingomonas* and *Nitrosospira* in Cd-contaminated soil, and thereby improving soil microbial community structure, finally promoting the growth of perilla seedlings.

## 1 Introduction

In recent years, with the development of engineering and agriculture technologies, heavy metal pollution in the ecological environment has been unsettling: the soil ecosystem balance was destroyed, causing adverse effects on organisms. Cadmium (Cd) is one of the toxic heavy metal elements; it can eventually enter the human body through soil, plants, and the food chain, posing a potential threat to human health (Burger, 2008; Leong and Chang, 2020).

Cd toxicity inhibited plant growth and development (Hu et al., 2013; Yang et al., 2021). Roots are predominant site for Cd accumulation in most plants (Wu et al., 2020). The cell wall is the first barrier to inhibit the entry of excess Cd into the cytoplasm and is the main site of Cd accumulation in the roots of many metals tolerant plants (Lux et al., 2011; Yang et al., 2018). Pectin in cell walls is a polysaccharide linked by α-1,4-galacturonic acid chain. Under Cd stress, galacturonic acid residues in pectin can be dissociated into negatively-charged carboxyl groups, meaning they can absorb Cd^2+^ and fix Cd in the cell wall, which reduces the damage of Cd toxicity to plants.

Soil microorganisms regulate soil structure and maintain soil fertility to promote the healthy growth of plants (Colin et al., 2017). Moreover, they are impactful on the cycles of soil carbon and nitrogen (Wang et al., 2021b), and are the main indicators of soil health. Cd pollution changes soil microorganism community structure and physiological activity, thus reducing the number of microbial communities and the soil enzyme activities (Wang et al., 2019; Liu et al., 2020; Pan et al., 2020).

Selenium (Se) is an essential trace element for organisms, and the organic form of Se is important for the body’s immune and reproductive systems, the accurate functioning of the thyroid and brain, and to enzyme activity within cells (Zhu et al., 2009; Punamiya et al., 2010; Natasha et al., 2018; Hossain et al., 2021). Se plays a role in the anti-lipid peroxidation in plants (Schiavon and Pilon-Smits, 2017; Wang et al., 2021a). Se supply increase the content of sugar and crude fiber in carrot fleshy roots, which ultimately increases the yield of carrots (Wang et al., 2006). Se supply increases the content of Se, Ca, and K, but decreased the content of Cd, Nickel (Ni), Lead (Pb), and other heavy metals in grapefruit (Zhu et al., 2017). Se also regulates stomata opening, thus increasing stomatal CO_2_ flux and the net photosynthetic rate (Pn) in rice (Zhang et al., 2014). Se supply decreased Cd absorption in plants by improving GSH-PX activity (Filek et al., 2008; Zembala et al., 2010; Lin et al., 2012; Ding et al., 2013; Feng et al., 2013).

Actinomycetes, fungi and bacteria are the three main groups of soil microorganisms. Changes in soil quality impact the population and activity of soil microorganisms (Griffiths and Philippot, 2013). Se supply alters the community structure and diversity of soil microorganisms (Wang et al., 2021a). Low concentrations of Se^4+^ or Se^6+^ increase the number and diversity of soil microorganisms, while high concentration showed an inhibitory effect (Rosenfeld et al., 2018).

Perilla (*Perilla frutescens* L.) is an annual herb of the family *Labiatae* (mints). At present, many countries have carried out large-scale commercial cultivation of perilla, and the economic value is substantial. However, several studies have shown that perilla is a Cd-accumulator (Wei et al., 2015; Xiang et al., 2019; Xiao et al., 2020). Therefore, reducing Cd accumulation in perilla is of great significance to food safety. Several studies indicated that Se can alleviate Cd toxicity and reduce Cd accumulation in plants (Filek et al., 2008; Zembala et al., 2010; Lin et al., 2012; Ding et al., 2013; Feng et al., 2013). These studies reveal the physiological effects of Se treatment on plants under Cd toxicity through analysis of Cd uptake, reactive oxygen species (ROS) accumulation, and nutrient balance, etc. Soil microorganisms significantly affect the growth and development by modulating nutritional status in plants (Gong et al., 2021). However, whether and how Se affects the growth of perilla under Cd stress by altering the soil microorganisms remain largely unclear. In this study, we investigated the effects of different Se treatments on perilla growth and soil microbial communities under Cd stress. These results provide a greater understanding of soil microbiome resiliency and the impacts of Cd as pollutant on the soil microbial communities, and provided a theoretical basis for the cultivation and food safety of perilla.

## 2 Materials and methods

### 2.1. Plant materials and growth conditions

The seeds of perilla (*Perilla frutescens* L.) cultivar ZB1 were washed and soaked in water for 4–6 hours and sowed in vermiculite. 7 days the seedlings were transferred to nutrient soil in a pot (25 cm in diameter and 30 cm in height) with or without Cd contamination, and grown in an incubator (16 h/8 h light/dark cycle) at 25°C. Four treatments were set up in the foliar Se spraying experiment, which were the control, 5 μM Na_2_SeO_3_ (Se), 10 mg/kg CdCl_2_ (Cd), and 5 μM Se + 10 mg/kg Cd. CdCl_2_ and Na_2_SeO_3_ of analytical purity grade is used in this experiment, with purity of 99% and >98% respectively. There were two seedlings in each pot. Se solution was sprayed once every two days after the perilla had been transplanted for 28 days; the same amount of deionized water was sprayed in the control and Cd treatment alone. After 36 days of treatment, the roots, leaves and soil were collected. Root Se application was divided into six treatments: control, 0.6 mg/kg Se, 1.2 mg/kg Se, 10 mg/kg Cd, 0.6 mg/kg Se + 10 mg/kg Cd and 1.2 mg/kg Se + 10 mg/kg Cd. Soil samples were divided into rhizospheric soil and non-rhizospheric soil. After 36 days of treatment, the root length, the fresh/dry weight of roots and leaves were measured. Each treatment was repeated four times.

### 2.2. Photosynthetic characteristics

The determination of photosynthetic characteristics was slightly changed according to the method of Gao et al. (2020). The photosynthetic data of the 2nd and 3rd leaves from the top of perilla seedlings were measured using a LI-6800 portable photosynthesis meter (LI-COR Company, USA) under natural light. The photosynthetic active radiation (PAR) intensity was set as 500 μmol·m^-2^·s^-1^, and the relative humidity (RH) was 50%. From 9 am to 11 am, the following photosynthetic parameters of perilla leaves were determined: Pn, stomatal conductance (Gs), intercellular CO_2_ concentration (Ci), and transpiration rate (Tr). The chlorophyll fluorescence parameters were measured by the photochemical quenching coefficient (qP), non-photochemical quenching coefficient (NPQ), actual photochemical efficiency of PSII reaction center (actual PSII), and apparent photosynthetic electron transport rate (ETR). Perilla seedlings were fully adapted to the dark for 30 min, F_0_ was measured. Then the maximum fluorescence yield of the dark-adapted state (F_m_) was detected under a saturated pulse, and the maximum photochemical efficiency [F_v_/F_m_, F_v_/F_m_=(F_m_-F_0_)/F_m_] of PSII was calculated. Followed by 40 s of darkness, 1 s of activated light, and one saturated pulse, the maximal fluorescence yield of the light-adapted state (F_m_’) was detected. Minimal fluorescence yield of the light-adapted state (F_0_’) was subsequently detected under far-red light. And then actual photochemical quantum yield [F_v_’/F_m_’, F_v_’/F_m_’=(F_m_’-F_0_’)/F_m_’] was calculated. The stomatal limitation value (Ls, Ls=1-Ci/Ca), water use efficiency (WUE, Pn/Tr), photosynthetic function limit value L (PFD) = 1-(F_v_’/F_m_’×qP)/0.83, and antenna heat dissipation (Hd, 1-F_v_’/F_m_’) were calculated from the measured data above. At the same time, the relative chlorophyll content of the perilla leaves was determined by SPAD (SPAD-502plus, Japan).

### 2.3. Cell wall compositions

The extraction of the root cell wall was slightly changed according to the method of Wang et al. (2017). The root system of the sample was frozen and ground in liquid nitrogen; next, 30 mL of 75% ethanol homogenate was centrifuged at 8000 rpm for 20 min at 4°C, and the supernatants was removed (this step was repeated 2-3 times), 100% acetone rinsed once, methanol: trichloromethane (1:1 v/v) of analytical purity grade rinsed once, methanol washed once, and the remaining precipitate was crude cell wall, which was dried or freeze-dried at 60°C and ground for use.

Appropriately 0.1 g crude cell wall samples were extracted with 2 mL deionized water 3 times at 100°C for 1 h each time, and this was done in order to ensure the separation of cell wall pectin and hemicellulose. The supernatant was collected for the determination of pectin content. The residual precipitate (CW_1_) was used for hemicellulose extraction. The precipitate (CW_1_) was extracted twice with 4% KOH containing 0.1% NaBH_4_ at room temperature for 12 h each time for a total of 24 h, and the supernatant was hemicellulose 1 (HC_1_). The residual precipitate (CW_2_) was used for hemicellulose 2 (HC_2_) extraction. The precipitate (CW_2_) was extracted twice with 24% KOH containing 0.1% NaBH_4_ for 12 h each time, and the supernatant was HC_2_.

The content of pectin was measured according to the method of Zhu et al. (2012). Briefly, 200 μL of the supernatant was removed for sampling, and 1mL 98% H_2_SO_4_-borax (0.0125%) was added. The mixture was then boiled in a 100°C water bath for 5 min. After cooling, 20 μL of 0.15% M-hydrodiphenyl was immediately added, and the liquid was mixed. Next, the mixture was left to stand for 20 min and the absorbance was measured at 520 nm. According to the method of Yang et al. (2011) and Zhu et al. (2013), the hemicellulose content was characterized by glucose content. 200 μL of the supernatant was removed for sampling, and then 10 μL 80% phenol was added. The resulting solution was then mixed with 1 mL 98% H_2_SO_4_, left to stand for 15 minutes, and boiled for 15 minutes at 100°C. The OD490 was determined after cooling.

### 2.4. Soil sample collection and treatment

For the soil sample collection and treatment, all appliances were sterilized and dried in advance. First, the mulch was removed from the soil, and then we gently shook off the upper soil on the aseptic paper, mixed it thoroughly, and sifted through 2 mm to form non-rhizospheric soil. After shaking the root, the large chunks of soil and the loose soil at the root of the perilla plant were removed, and the residual soil was sifted from the root with a sterile brush to form the rhizospheric soil. The non-rhizospheric soil and rhizospheric soil were put into a 10 mL sterilized tube with a sterilized key, labeled and sealed with film, frozen in sample bags, and then stored in a refrigerator at -40°C. Each process was repeated 5 times.

### 2.5. DNA extract and PCR products mixing and purification

For the microbial genome extraction and sequencing in rhizospheric soil and non-rhizospheric soil, high-throughput sequencing was completed by Hangzhou Lianchuan Biotechnology Co., Ltd. DNA extraction strictly followed the instructions of E.Z.N.A.^®^ Soil DNA genomic DNA extraction kit. The purity and concentration of the extracted DNA samples were detected by agarose gel electrophoresis, and the extracted DNA was quantified with an ultraviolet spectrophotometer. Subsequently, the V3–V4 region of the sequence was sequenced with primers 341F (5’-CCTACGGGNGGCWGCAG-3’) and 805R (5’-GACTACHVGGGTATCTAATCC-3’), and polymerase chain reaction (PCR) amplification was carried out during the sequencing process. The PCR products were confirmed by 2% agarose gel electrophoresis. In order to eliminate the possibility of false-positive PCR results, as negative control, ultra-pure water instead of sample solution was used during the entire process of DNA extraction. PCR products were purified by AMPure XT beads (Beckman Coulter Genomics, Danvers, MA, USA) and quantified by Qubit (Invitrogen, USA).

### 2.6. Library preparation and sequencing

The size and number of amplified sub-libraries were evaluated with the library quantitative kits of Agilent 2100 BioAnalyzer (Agilent, USA) and Illumina (Kapa Biosciences, Woburn, MA, USA), respectively. The library was sorted on the NovaSeq PE250 platform. Samples were sequenced on the Illumina NovaSeq platform and provided by LC-Bio, as recommended by the manufacturer. According to the unique bar code of the sample, a paired end sequence was assigned to the sample, and the barcode and primer sequences introduced into the database were removed. According to fqtrim (v0.94), the quality of the original read data was filtered under specific filtering conditions to obtain high-quality clean tags. The chimeric sequences were filtered by VSEARCH software (v2.3.4).

### 2.7. Statistical analysis

The physiological experiment data were plotted by Excel 2010 software and were analyzed by LSD and Duncan in SPSS 20.0. The analysis was carried out at the level of p < 0.05. The data were expressed in the form of mean ± standard error. Using DADA2 for extraction, the feature table and feature sequence were obtained. Alpha and beta diversity were calculated by normalizing to the same random sequence. Then, according to the SILVA (Release 132) classifier, the feature abundance was normalized using the relative abundance of each sample. Alpha diversity was used to analyze the complexity of species diversity in samples. Five indicators, including the Chao1, Observed Species, Good Coverage, Shannon, and Simpson Indexes, were used to analyze the complexity of species diversity. All of the indicators in the sample were calculated by QIIME2. Beta diversity was calculated by QIIME2 and plotted using the R package. Blast was used for sequence alignment, and each representative sequence was annotated with the SILVA database. Based on the Feature Abundance Table, the weighted Unifrac distances was calculated, and principal coordinates analysis (PCoA) was performed on the soil bacterial communities of the 4 treatments, the differences was estimated by the functions of *vegan* packages in R (v3.5.2) (Oksanen et al., 2014).

## 3 Results

### 3.1. Effects of selenium on seedling growth in *Perilla frutescens*

#### 3.1.1 Foliar spraying selenium

Cd treatment did not cause significant phenotypic changes in perilla. Compared with the 10 mg/kg Cd treatment alone, foliar spraying with 5 μM Se increased the leaf dry weight, root fresh weight and dry weight, and root length increased by 13.19%, 38.22%, 42.31% and 0.61%, respectively, in Cd-treated plants (**Table S1**), indicating foliar spraying with Se alleviated Cd toxicity in perilla.

#### 3.1.2 Root exposure selenium

After 0.6 mg/kg Se treatment in soil, the leaf dry weight, root fresh weight and dry weight increased by 2.87%, 3.16%, and 6.18%, respectively. While, after 1.2 mg/kg Se treatment in soil, the leaf fresh weight and dry weight, and root length increased by 20.62%, 12.98% and 8.74%, respectively (**Table S2**).

Compared with the 10 mg/kg Cd treatment alone, root exposure of 0.6 mg/kg Se increased the leaf fresh weight and dry weight, root fresh weight and dry weight by 13.37%, 14.29%, 14.77% and 14.67%, respectively. Moreover, root exposure of 1.2 mg/kg Se increased the leaf fresh weight and dry weight, root fresh weight and dry weight by 2.27%, 26.48%, 19.83% and 36.19%, respectively (**Table S2**). Taken together, these results indicated that both foliar supply and root exposure Se protected plant from Cd toxicity and promoted plant growth under Cd stress.

### 3.2. Effects of selenium on photosynthesis efficiency and water absorption in *Perilla frutescens*

#### 3.2.1 Foliar spraying selenium

After 10 mg/kg Cd treatment, the chlorophyll content was 0.77% lower than the control (**Figure S1**). Foliar spraying with 5 μM Se increased the relative chlorophyll content by 9.92% compared with the untreated control. Compared with the 10 mg/kg Cd treatment alone, foliar spraying with 5 μM Se increased the chlorophyll content by 7.31% (**Figure S1**).

After 10 mg/kg Cd treatment, the ΦPSII and ETR decreased by 14.31% and 14.30%, respectively (**Figure S1**). Foliar spraying with 5 μM Se increased the ΦPSII and ETR by 26.89% and 26.89%, respectively (**Figure S1**). Compared with the 10 mg/kg Cd treatment alone, foliar spraying with 5 μM Se increased the ΦPSII and ETR by 38.17% (**Figure S1**).

#### 3.2.2 Root exposure selenium

**Figure 1:**
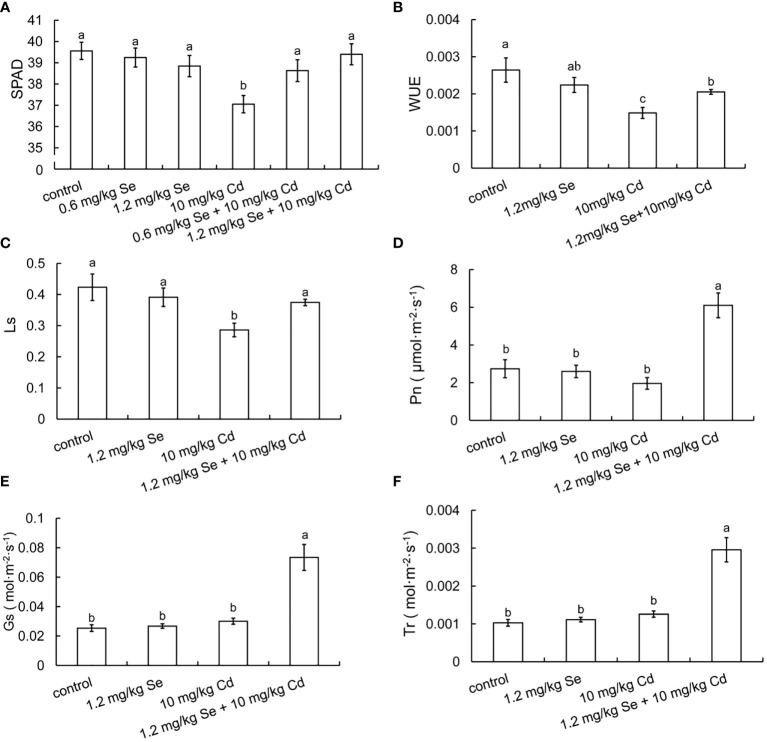
Effects of root exposure of Se on photosynthetic efficiency of perilla seedlings under Cd stress (10 mg/kg). **(A)** SPAD; **(B)** water use efficiency; **(C)** stomatal limit value; **(D)** net photosynthetic rate; **(E)** stomatal conductance; **(F)** transpiration rate. The different letters indicate significantly different values (P < 0.05 according to Duncan’s test).

Compared with the 10 mg/kg Cd treatment alone, root exposure of 0.6 or 1.2 mg/kg Se increased the chlorophyll contents by 4.26% and 6.33%, respectively (**Figure 1**). Root exposure to 1.2 mg/kg Se decreased WUE and Ls by 43.71% and 32.39%, respectively. Compared with the 10 mg/kg Cd treatment alone, root exposure to 1.2 mg/kg Se increased Pn, Gs, Tr, WUE and Ls by 211.17%, 144.21%, 135.03%, 38.12% and 30.80%, respectively (**Figure 1**). These results collectively indicated that both foliar supply and root exposure Se increased photosynthesis and water use efficiency in plants, thereby improving Cd tolerance in plants.

### 3.3. Effects of root exposure to selenium on cell wall components in *Perilla frutescens*

**Figure 2:**
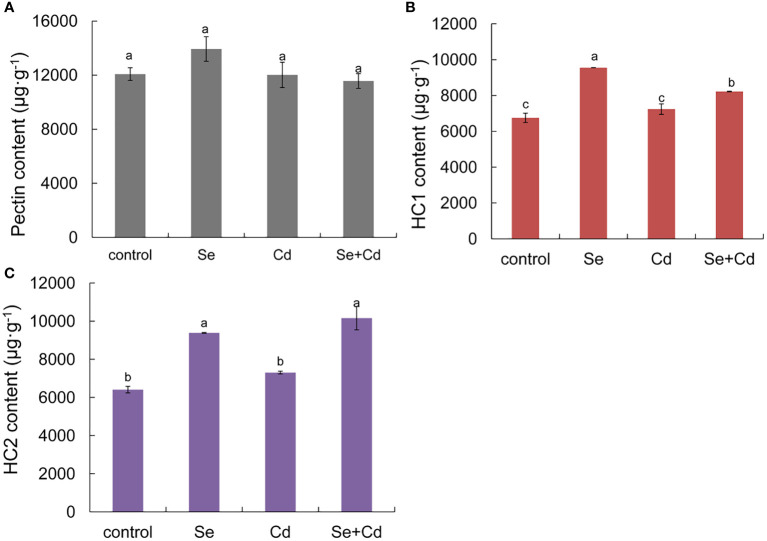
Effects of root exposure of Se (1.2 mg/kg) on root cell wall components of perilla under Cd stress (10 mg/kg). **(A)** pectin content; **(B)** hemicellulosic 1**(C)** hemicellulosic 2. The different letters indicate significantly different values (P < 0.05 according to Duncan’s test).

As shown in **Figure 2**, root exposure to 1.2 mg/kg Se increased the contents of pectin, HC_1_, and HC_2_ by 15.40%, 41.51% and 46.44%, respectively. Compared with the 10 mg/kg Cd treatment alone, root exposure to 1.2 mg/kg Se increased the content of HC_1_ and HC_2_ by 13.60% and 39.11%, respectively. Hemicellulose, which is distributed on the surface of cellulose microfibrils, has a role in fixing heavy metals. Ma et al. (2015) determined the content of Cd in root cell walls and found that the bound Cd in hemicellulose was higher than that in pectin. Increased accumulation of the hemicellulose component is helpful for fixing Cd in the cell wall and restricting the transportation of Cd into the cell, thus improving Cd toxicity tolerance in perilla.

### 3.4. Effects of root exposure to selenium on soil microbial community structure in *Perilla frutescens*

#### 3.4.1 Effects on the biological characteristics of the rhizosphere soil

As shown in **Table S3**, the sample data after sequencing was statistically summarized. The results show that the sequencing data are abundant. The effective data account for 100.00%; Q20 and Q30 are above 96.40% and 89.80%, respectively, and the GC content is more than 56.58%, which ensures the reliability of the follow-up analysis results. The alpha diversity analysis of soil microbiomes showed that the application of Se and Cd did not significantly change the diversity of microorganisms or the richness of communities in rhizosphere soil samples. In addition, the Goods Coverage Index for all samples is above 99%, indicating that the sequencing depth is sufficient (**Table S4**).

As shown in **Figure S2A**, except for Se treatment alone, the four duplicates in the remaining treatments were not clustered in the same quadrant, indicating a large variation within the group. PCoA analysis revealed that the dominant factor affecting the soil bacterial community structure was obvious. An Adonis analysis further showed that Se and Cd treatment had a significant impact on the soil bacterial community structure (R^2^ = 0.32, *P* = 0.002), supporting the test’s availability.

**Figure 3:**
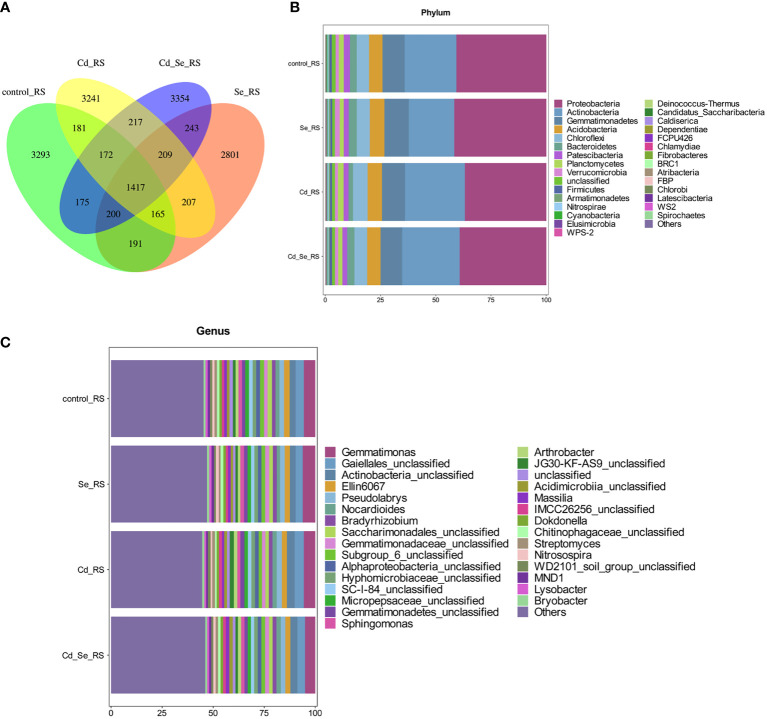
Effects of root exposure to Se (1.2 mg/kg) on rhizosphere soil microbial community structure in perilla under Cd stress (10 mg/kg). **(A)** Venn plot; **(B)** Histogram of species abundance at phylum level; **(C)** Histogram of species abundance at genera level.

Additional Venn diagram showed the number of features common to or unique to the soil microbiomes of different treatment groups, and then the similarity, overlap, and specificity of the structure of the microbiomes between treatment groups were analyzed. As shown in **Figure 3A**, the total number of feature strips in each treated soil was 1,417, while the number of feature strips specific to the control, Se-, Cd-, and Se+Cd-treated soil bacterial communities was 3,293, 2,801, 3,241, and 3,354, respectively. The results indicated that Se+Cd treatment showed the most complex rhizosphere soil bacterial community structure, and that had the highest degree of specificity. The results also revealed that Se and Cd treatment reduced the specificity of the soil bacterial community structure.

We then analyzed the relative abundance of the top 30 phylum of the bacteria, of which 9 had an average relative abundance of more than 1%; the largest were *Proteobacteria*, *Actinobacteria*, *Gemmatimonades*, *Chloroflexi*, *Acidobacteria*, *Bacteroidetes*, *Patescibacteria*, *Planctomycetes*, and *Verrucomicrobia.* The sequences belonging to *Proteobacteria*, *Acidobacteria*, *Gemmatimonadetes*, *Chloroflexi* and *Acidobacteria* account for more than 80% of the total sequence, and these microorganisms were the dominant populations (**Figure 3B**). We subsequently analyzed the relative abundance of the top 30 genera of the bacteria, of which 24 had an average relative abundance of more than 1%. *Gematimonas*, *Gaiellales_unclassified*, *Actinobacteria_unclassified*, *Ellin6067* and *Pseudolabrys* have relative abundances greater than 2%, and these microorganisms are the dominant populations (**Figure 3C**).

**Figure 4:**
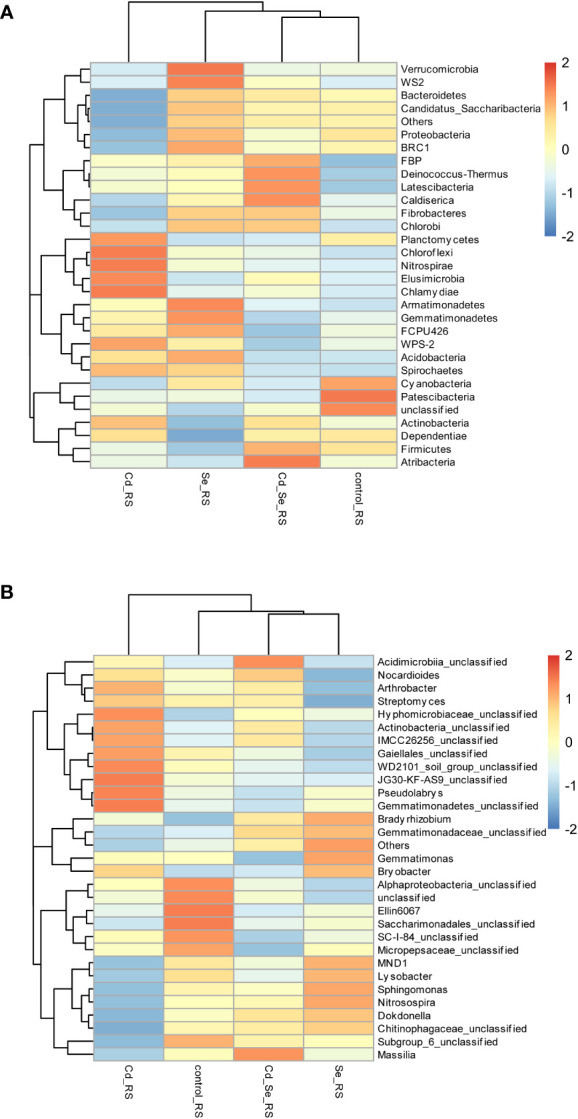
Effects of root exposure to Se (1.2 mg/kg) on rhizosphere soil species abundance in perilla under Cd stress (10 mg/kg). **(A)** Phylum level; **(B)** Genus level.

Compared to the control, the main increased phylums of bacteria under Cd treatment were *FBP*, *Deinococcus-Thermus*, *Latescibacteria*, *Planctomycetes*, *Chloroflexi*, *Nitrospirae*, *Elusimicrobia*, *Chlamydiae*, *WPS-2*, *Spiroetchaes* and *Actinobacteria*. The main decreased phylums of bacteria under Cd treatment were *Bacteroidetes*, *Candidatus_Saccharibacteria*, *Cyanobacteria* and *Patescibacteria*. Additionally, under Se treatment, *Verrucomicrobia*, *WS2*, *BRC1*, *Armatimonadetes*, *Gemmatimonadetes*, *FCPU426*, *Acidobacteria*, *Spirochaetes*, *Bacteroidetes*, *Candidatus_Saccharibacteria* and *Proteobacteria* increased, whereas *Cyanobacteria*, *Patescibacteria*, *Planctomycetes*, *Firmicutes*, *Dependentiae* and *Actinobacteria* decreased. Furthermore, the abundance of *WS2*, *FBP*, *Deinococcus-Thermus*, *Latescibacteria*, *Caldiserica*, *Fibrobacteres*, *Chlorobi*, *Firmicutes*, *Atribacteria* increased, whereas *Cyanobacteria*, *Patescibacteria*, and *Planctomycetes* decreased in Cd+Se treatment. Compared with Cd treatment alone, the abundance of *FBP*, *Deinococcus-Thermus*, *Latescibacteria*, *Caldiserica*, *Fibrobacteres*, *Chlorobi*, *Atribacteria*, *Bacteroidetes* and *Candidatus_Saccharibacteria* increased in the soil of Cd+Se treatment; however, it decreased the abundance of *Planctomycetes*, *Chloroflexi*, *Nitrospirae*, *Elusimicrobia*, *Chlamydiae*, *WPS-2*, *Spirochaetes* (**Figure 4A**).

At the phylum level, Cd treatment alone increased the abundance of *Actinobacteria* and *Planctomycetes*, and it decreased the abundance of *Proteobacteria, Bacteroidetes* and *Candidatus_Saccharibacteria* in the rhizosphere soil. Se treatment also increased the abundance of *Proteobacteria*, *Bacteroidetes* and *Candidatus_Saccharibacteria*, while it decreased the abundance of *Actinobacteria* and *Planctomycetes* in the rhizosphere soil. Compared with Cd treatment alone, root application of exogenous Se under Cd stress increased the abundance of *Proteobacteria*, *Bacteroidetes* and *Candidatus_Saccharibacteria* in the rhizosphere soil; it also decreased the abundance of *Actinobacteria* and *Planctomycetes*. The results showed that Se inhibited the growth of *Actinobacteria* and *Planctomycetes*, while it promoted the growth of *Proteobacteria*, *Bacteroidetes* and *Candidatus_Saccharibacteria* (**Figure 4A**).

Compared with the control, the main genus of bacteria that increased in Cd treatment group were *Bryobacter*, *Arthrobacter*, *Streptomyces*, *Hyphomicrobiaceae_unclassified*, *Actinobacteria_unclassified*, *IMCC26256_unclassified*, *Gaiellales_unclassified*, *WD2101_soil_group_unclassified*, *JG30-KF-AS9_unclassified*, *Pseudolabrys*, *Gemmatimonadetes_unclassified* (**Figure 4B**). The main genus of bacteria that decreased in Cd treatment were *MND1*, *Sphingomonaz*, *Nitrosospira*, *Dokdonella*, *Chitinophagaceae_unclassified*, *Alphaproteobacteria_unclassified*, *Ellin6067*, *Saccharimonadales_unclassified*, *SC-I-84_unclassified*, *Micropepsaceae_unclassifie*. The main genus of bacteria that increased in Se treatment were *MND1*, *Sphingomonas*, *Nitrosospira*, *Dokdonella*, *Chitinophagaceae_unclassified*, *Gemmatimonas*, *Bradyrhizobium*, *Gemmatimonadaceae_unclassified*. The main genus of bacteria that decreased in Se treatment were *Alphaproteobacteria_unclassified*, *Ellin6067*, *Saccharimonadales_unclassified*, *SC-I-84_unclassified*, *Micropepsaceae_unclassified*, *Subgroup_6_unclassified*, *Actinobacteria_unclassified*, *Gaiellales_unclassified*, *Nocardioides*, *Arthrobacter*, *Streptomyces*. The main genus of bacteria that decreased in Cd+Se treatment were *Massilia*, *Acidimicrobiia_unclassified*, *Nocardioides* (**Figure 4B**).

The main genus of bacteria that increased in Cd+Se treatment were *Alphaproteobacteria_unclassified*, *Ellin6067*, *Saccharimonadales_unclassified*, *SC-I-84_unclassified*, *Micropepsaceae_unclassified*, *Subgroup_6_unclassified*. Compared with Cd treatment alone, the abundance of *Massilia*, *MND1*, *Sphingomonas*, *Nitrosospira*, *Dokdonella*, *Chitinophagaceae_unclassified*, *Subgroup_6_unclassified*, *Acidimicrobiia_unclassified* and *Gemmatimonadaceae_unclassified* increased in Cd+Se treatment; and the abundance of *SC-I-84_unclassified*, *Micropepsaceae_unclassified*, *Bryobacter*, *Arthrobacter*, *Streptomyces*, *Gemmatimonas*, *Hyphomicrobiaceae_unclassified*, *Actinobacteria_unclassified*,*IMCC26256_unclassified*, *Gaiellales_unclassified*, *WD2101_soil_group_unclassified*, *JG30-KF-AS9_unclassified*, *Pseudolabrys*, *Gemmatimonadetes_unclassified* were decreased in Cd+Se treatment (**Figure 4B**).

The abundance of *Actinobacteria_unclassified*, *Gaiellales_unclassified*, *Arthrobacter* and *Streptomyces* in the rhizosphere soil of Cd treatment increased at the genus level but it decreased in the rhizosphere soil Se treatment. Compared with Cd treatment alone, root application of Se under Cd stress increased the abundance of *MND1*, *Sphingomonas*, *Nitrosospira*, *Dokdonella* and *Chitinophagaceae_unclassified* in the rhizosphere soil but decreased the abundance of *Actinobacteria_unclassified*, *Gaiellales_unclassified*, *Arthrobacter* and *Streptomyces* in the rhizosphere soil. Taken together, Se inhibited the growth of *Actinobacteria_unclassified*, *Gaiellales_unclassified*, *Arthrobacter* and *Streptomyces* while it promoted the growth of *MND1*, *Sphingomonas*, *Nitrosospira*, *Dokdonella*, and *Chitinophagaceae_unclassified* (**Figure 4B**).

#### 3.4.2 Effects on the biological characteristics of the non-rhizosphere soil

As shown in **Table S5**, the effective data of each sample after sequencing was statistically summarized. The results showed that the amount of sequencing data was abundant; the valid data accounted for 100.00%; Q20 and Q30 were above 95.27% and 87.11%, respectively, and the GC content was above 56.45%, thus ensuring the reliability of subsequent analysis results.

**Table S6** shows that Se and Cd application were not significant on the Shannon index, Simpson index, Observed Species Index, or Chao1 Index of bacterial community in the non-rhizosphere soil. The Alpha diversity analysis of the soil microbial community also showed that the application of Se and Cd did not significantly change the microbial diversity and community richness in the non-rhizosphere soil samples. In addition, the Good Coverage Index of all samples was 100%, indicating that the sequencing depth was sufficient.

**Figure 5:**
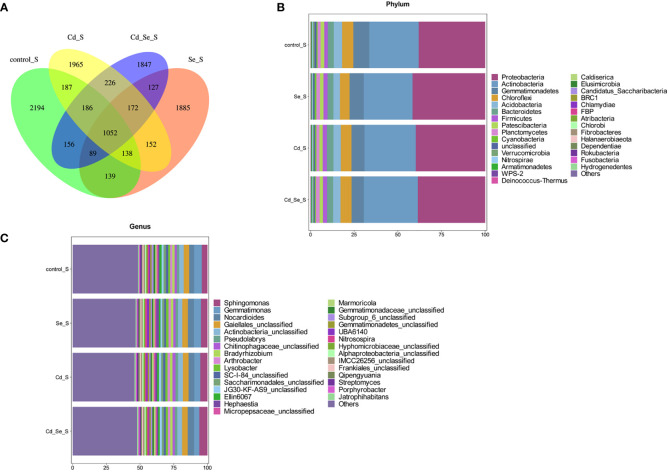
Effects of root exposure to Se (1.2 mg/kg) on non-rhizosphere soil microbial community structure in perilla under Cd stress (10 mg/kg). **(A)** Venn plot; **(B)** Histogram of species abundance at phylum level; **(C)** Histogram of species abundance at genera level.

Except for the Se treatment alone, the repeats in the other treatments were not clustered in the same quadrant, indicating that there was a large variation within the group (**Figure S2B**). There was a significant difference between the control and Cd treatments. This in turn indicates that the dominant factors affecting soil bacterial community structure were obvious. The total number of features in the bacterial community in each treatment was 1,052, whereas the number of unique features in the control, Se, Cd, and Se+Cd treatments was 2,194, 1,885, 1,965 and 1,847, respectively (**Figure 5A**). The results revealed that the soil bacterial community structure of the untreated control group was the most complex and specific, and that the specificity of soil bacterial community structure was reduced by Se, Cd or their combination.

The relative abundance of the top 30 phylums of the bacteria was shown in **Figure 5B**, of which 9 phylums had an average relative abundance of more than 1%, including *Proteobacteria*, *Actinobacteria*, *Gemmatimonadetes*, *Chloroflexi*, *Acidobacteria*, *Bacteroidetes*, *Firmicutes*, *Patescibacteria* and *Planctomycetes*. Among them, the sequences belonging to *Proteobacteria*, *Actinobacteria*, *Gemmatimonadetes*, *Chloroflexi* and *Acidobacteria* accounted for more than 80% of the total sequences, and these microorganisms were the dominant populations. The relative abundance of the top 30 genus of the bacteria was shown in **Figure 5C**, of which 16 phylums had an average relative abundance of more than 1%, including *Gemmatimonas*, *Gaiellales*_*unclassified*, *Actinobacteria_unclassified*, *Sphingomonas* and *Nocardioides*.

**Figure 6:**
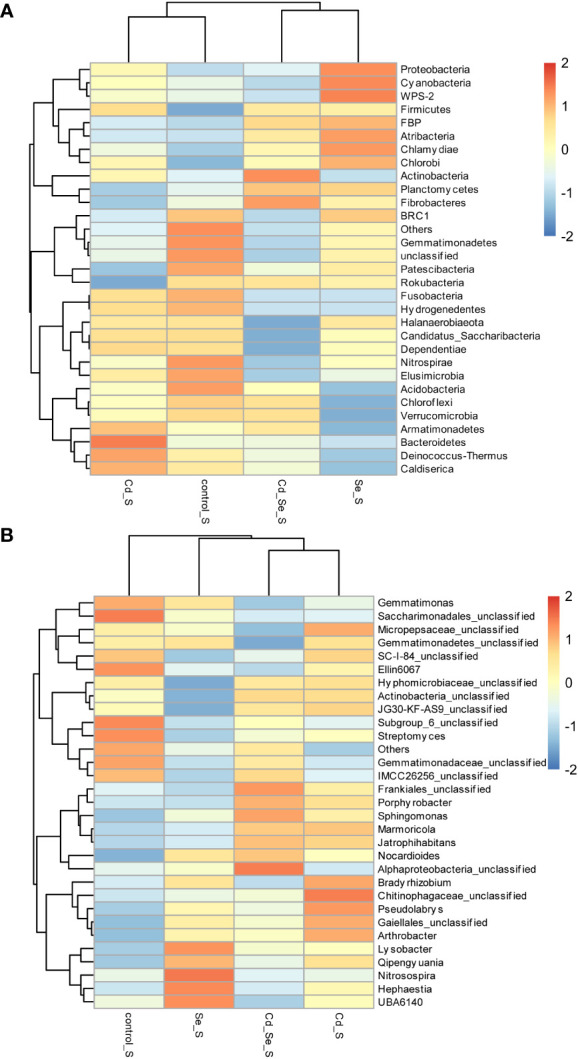
Effects of root exposure to Se (1.2 mg/kg) on non-rhizosphere soil species abundance in perilla under Cd stress (10 mg/kg). **(A)** Phylum level; **(B)** Genus level.

Compared to the control group, the abundance of the phylums of bacteria that increased under Cd treatment included *Firmicutes*, *Bacteroidetes*, *Proteobacteria*, *Cyanobacteria*, *Chlamydiae*, *Chlorobi*, *Actinobacteria*, *Armatimonadetes*, *Deinococcus-Thermus* and *Caldiserica*, while abundance of *Planctomycetes*, *Fibrobacteres*, *BRC1*, *Gemmatimonadetes*, *Patescibacteria*, *Rokubacteria*, *Nitrospirae*, *Elusimicrobia*, *Acidobacteria*, *Chloroflexi* and *Verrucomicrobia* decreased. Se treatment increased the abundance of *Proteobacteria*, *Cyanobacteria*, *WPS-2*, *Firmicutes*, *FBP*, *Atribacteria*, *Chlamydiae*, *Chlorobi*, *Planctomycetes*, and *Fibrobacteres* while it decreased *Gemmatimonadetes*, *Patescibacteria*, *Fusobacteria*, *Hydrogenedentes*, *Nitrospirae*, *Elusimicrobia*, *Acidobacteria*, *Chloroflexi*, *Armatimonadetes*, *Verrucomicrobia*, *Bacteroidetes*, *Deinococcus-Thermus*, *Caldiserica*. Cd+Se treatment increased the abundance of *Firmicutes*, *FBP*, *Actinobacteria*, *Planctomycetes* and *Fibrobacteres*, while it decreased the abundance of *BRC1*, *Gemmatimonadetes*, *Patescibacteria*, *Fusobacteria*, *Hydrogenedentes*, *Nitrospirae*, *Elusimicrobia* and *Acidobacteria*. The abundance of bacteria increased after Cd+Se treatment including *Actinobacteria*, *Planctomycetes*, *Atribacteria*, *Chloroflexi*, *Verrucomicrobia*, *Rokubacteria* and *Fibrobacteres*, while the abundance of bacteria decreased after Cd+Se treatment including *Bacteroidetes*, *WPS-2*, *Fusobacteria*, *Hydrogenedentes*, *Armatimonadetes*, *Deinococcus-Thermu*s, *Caldiserica*, *Elusimicrobia*, *Candidatus_Saccharibacteria*, *Proteobacteria*, *Cyanobacteria*, *Halanaerobiaeota*, *Dependentiae* (**Figure 6A**).

At the phylum level, Cd treatment increased the abundance of *Armatimonadetes*, *Deinococcus-Thermus*, and *Caldiserica*, and it decreased the abundance of *Fibrobacteres* in non-rhizosphere soil. Se treatment increased the abundance of *Fibrobacteres* but decreased the abundance of *Armatimonadetes*, *Deinococcus-Thermus* and *Caldiserica.* Compared with Cd treatment alone, Se increased the abundance of *Fibrobacteres* and decreased the abundance of *Armatimonadetes*, *Deinococcus-Thermus* and *Caldiserica* in the non-rhizosphere soil under Cd stress. Se inhibited the growth *of Armatimonadetes*, *Deinococcus-Thermus*, and *Caldiserica* while it promoted the growth of *Fibrobacteres* (**Figure 6A**).

Compared to the control group, the genus of bacteria that increased after Cd treatment were *Micropepsaceae_unclassified*, *Actinobacteria_unclassified*, *JG30-KF-AS9_unclassified*, *Frankiales_unclassified*, *Porphyrobacter*, *Sphingomonas*, *Marmoricola*, *Jatrophihabitans*, *Nocardioides*, *Bradyrhizobium*, *Chitinophagaceae_unclassified*, *Pseudolabrys*, *Gaiellales_unclassified*, *Arthrobacter* and *Qipengyuania*, while the abundance of *Gemmatimonadaceae_unclassified*, *Gemmatimonas*, *Saccharimonadales_unclassified*, *Ellin6067*, *Subgroup_6_unclassified*, *Streptomyces*, *IMCC26256_unclassified* were decreased. The abundance of bacteria that increased after Se treatment were *Nocardioides*, *Bradyrhizobium*, *Pseudolabrys*, *Gaiellales_unclassified*, *Arthrobacter*, *Qipengyuania*, *Lysobacter*, *Nitrosospira*, *Hephaestia* and *UBA6140* while the abundance of *JG30-KF-AS9_unclassified*, *Streptomyces*, *IMCC26256_unclassified*, *Gemmatimonas*, *Ellin6067*, *Gemmatimonadaceae_unclassified*, *Saccharimonadales_unclassified*, *Subgroup_6_unclassified*, *SC-I-84_unclassified*, and *Hyphomicrobiaceae_unclassified* were decreased (**Figure 6B**).

The abundance of bacteria that increased under Cd+Se treatment were *Frankiales_unclassified*, *Porphyrobacter*, *Sphingomonas*, *Marmoricola*, *Jatrophihabitans*, *Nocardioides* and *Alphaproteobacteria_unclassified*; in comparison, the abundance of *Gemmatimonas*, *SC-I-84_unclassified*, *Saccharimonadales_unclassified*, *Ellin6067*, *Streptomyces*, *Micropepsaceae_unclassified*, *Gemmatimonadetes_unclassified*, *Subgroup_6_unclassified* and *Gemmatimonadaceae_unclassified* were decreased. Compared to Cd treatment alone, Cd+Se treatment increased bacteria numbers, including *Frankiales_unclassified*, *Gemmatimonadaceae_unclassified*, *Subgroup_6_unclassified*, *IMCC26256_unclassified*, *Alphaproteobacteria_unclassified*, and *Sphingomonas*, etc. However, Cd+Se treatment decreased the rates of *Micropepsaceae_unclassified*, *Arthrobacter*, *Qipengyuania*, *Ellin6067*, *Bradyrhizobium*, *Pseudolabrys*, *Gaiellales_unclassified*, *Gemmatimonadetes_unclassified* and *Chitinophagaceae_unclassified* (**Figure 6B**).

At the genus level, the abundance of *JG30-KF-AS9_Unclassified, Micropepsaceae_unclassified*, and *Actinobacteria_unclassified* were increased under Cd treatment, while the abundance of *JG30-KF-AS9_Unclassified*, *Micropepsaceae_unclassified* and *Actinobacteria_unclassified* were decreased under Se treatment. Compared with Cd alone, the abundance of *JG30-KF-AS9_Unclassified, Micropepsaceae_unclassified*, and *Actinobacteria_unclassified* decreased after Se application under Cd stress. Se inhibited the growth of *JG30-KF-AS9_unclassified*, *Micropepsaceae_unclassified* and *Actinobacteria_unclassified* (**Figure 6B**).

#### 3.4.3 Comparison of the rhizosphere soil and the non-rhizosphere soil

Venn diagram analysis showed that the bacterial community structure in the rhizosphere soil was more complex and specific than that in the non-rhizosphere soil (**Figure S3**).

**Figure 7:**
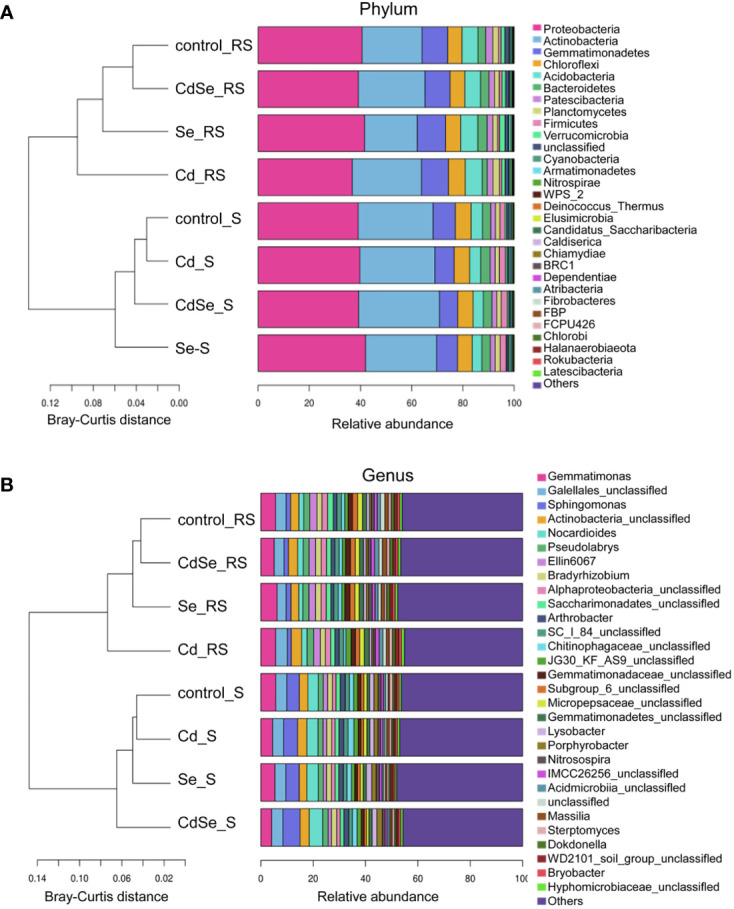
Effects of root exposure to Se (1.2 mg/kg) on soil species abundance in perilla under Cd stress (10 mg/kg). **(A)** Phylum level; **(B)** Genus level.

Investigation of the TOP30 of phylum of bacteria revealed that 8 of which had an average relative abundance of more than 1% in both the rhizosphere soil and non- rhizosphere soil, including *Proteobacteria*, *Actinobacteria*, *Gemmatimonadetes*, *Chloroflexi*, *Acidobacteria*, *Bacteroidetes*, *Patescibacteria*, and *Planctomycetes* (**Figure 7A**). The relative abundance of *Gemmatimonadetes*, *Acidobacteria*, *Patescibacteria*, and *Verrucomicrobia* in the rhizosphere soil was higher than those in the non-rhizosphere soil; however, the opposite pattern is true for *Actinobacteria* and *Firmicutes*. Cluster analysis revealed that there were significant differences in the distribution of bacteria between the rhizosphere soil and the non-rhizosphere soil.

Investigation of the TOP30 of genus of bacteria revealed that 3 of which had an average relative abundance of more than 2% in both the rhizosphere soil and non- rhizosphere soil, including *Gemmatimonas*, *Gaiellales_unclassified* and *Actinobacteria_unclassified* (**Figure 7B**). The average relative abundance of *Gemmatimonas*, *Gaiellales_unclassified*, *Actinobacteria_unclassified*, *Ellin6067*, *Pseudolabrys* in the rhizosphere soil was more than 2%, which respectively accounted for 5.00%–6.15%, 3.47%–4.50%, 2.82%–3.78%, 2.47%–2.75%, and 2.23%–2.52%. While, *Sphingomonas*, *Gemmatimonas*, *Nocardioides*, *Gaiellales_unclassified* and *Actinobacteria_unclassified* had an average relative abundance of over 2% in the non- rhizosphere soil, which respectively accounted for 4.20%–6.03%, 3.95%–5.75%, 3.59%–4.67%, 4.10%–4.24%, and 3.07%–3.51%. The relative abundance of *Ellin6067* and *Pseudolabrys* in the rhizosphere soil was higher than that in the non- rhizosphere soil, whereas *Sphingomonas* and *Nocardioides* were less abundant in the rhizosphere soil than that in the non- rhizosphere soil.

## 4 Discussion

As one of the most toxic heavy metal elements, Cd usually exists as Cd^2+^ in the soil and is considered the most toxic form of Cd (Riaz et al., 2021). Cd exists in soil as exchangeable (bioavailable and mobile form), organic, oxide and carbonate (fixed and immobile form) (Hussain et al., 2020). As desorption from soil solid phase, Cd is released into the soil solution, which enters into the roots *via* mass flow or diffusion (Shahid et al., 2017). Soil pH significantly influences metal phytoavailability in soil (Rafiq et al., 2014). In alkaline solutions, Cd is present as CdHCO^3+^ or CdCO_3_, which cannot be uptake by plant roots, and contribute to its less bioavailable. With the decrease in soil pH, the adsorption of Cd^2+^ by soil constituents decreased, and Cd^2+^ mobility and availability increased. The transformation of Cd forms increases from stable structure (such as carbonates, Fe, and Mn oxide bound structure) to effective bioavailable structure (such as exchangeable structure) (Li et al., 2013), thereby resulting in increased dissolvability of Cd^2+^ in soil and arrangement leading to higher uptake by plants, posing a greater threat to plants. (Hu et al., 2016).

Cd toxicity inhibits photosynthesis and respiration. The SPAD-chlorophyll, chlorophyll a, and chlorophyll b activities of lettuce leaves decreased in cadmium-contaminated soils (Ma et al., 2022), which might be related to the detrimental effects of Cd in contaminated soils (Sehrish et al., 2019). Our results revealed that foliar spraying with Se improved the levels of ΦPSII and ETR (**Figure S1**), indicating that Se protected the photosynthetic system under Cd toxicity by alleviating Cd-mediated reduction of chlorophyll content and increasing the photochemical activity of the PSII reaction center and the photochemical efficiency in perilla leaves.

Cell wall is composed of cellulose, hemicellulose, pectin, and structural glycoproteins which contain large negatively charged functional groups, such as, carboxyl, hydroxyl, amino, and phosphate (Berni et al., 2018; Vaahtera et al., 2019). Pectin and hemicellulose play a role in reducing heavy metal uptake in root cells by detaining heavy metal ions outside the cells (Ma et al., 2015). Previous studies have demonstrated that the pectin content in the root cell wall was increased under Cd toxicity in plants (Wu et al., 2020). Zhu et al. (2013) found that Cd could be fixed in HC_1_ of cell wall in *Arabidopsis* roots, and exogenous NAA decreased Cd uptake by increasing the content of HC_1_ in roots. Our results showed that Cd toxicity slightly increased the hemicellulose content, and Se treatment increased the contents of pectin, HC_1_, and HC_2_ in perilla roots (**Figure 2**). Moreover, root application of Se increased the contents of HC_1_ and HC_2_ in Cd-treated perilla seedlings (**Figure 2**). These results collectively indicated that Se reduced Cd accumulation by modulating the components of cell wall in perilla roots.

Both of Se and Cd altered the soil microbial community structure, but they did not affect soil bacterial community diversity (**Tables S4**, **S6**; **Figure S2**). The relative abundance of *Proteobacteria* and *Bacteroidetes* decreased under Cd toxicity, whereas they increased after applying Se (**Figures 4A** and **6A**). *Proteobacteria*, which is widely found in aquatic environments, is likely to adapt to a variety of plant rhizosphere microenvironments. It can not only promote the degradation of organic matter and the biological cycle of mineral elements, but it can also participate in the biological transformation of heavy metal ions (Rudi et al., 2010). *Bacteroidetes* are the main mineralizers of soil organic carbon, these microorganisms play a role in the degradation of macromolecular organic matter, sludge hydrolysis and acid production in the soil (Tonanzi et al., 2021). *Actinobacteria* is one of plant growth-promoting microorganisms (Chaurasia et al., 2018). Consistent with the previous results (Bamborough and Cummings, 2009; Gomes et al., 2010), we found that Cd pollution increased the relative abundance of *Actinobacteria* in the rhizosphere soil. *Fibrobacteres* can degrade cellulose, thus having a positive effect on the degradation of plant tissues in the soil and improving the soil microenvironment (Ransom-Jones et al, 2014). Cd pollution reduced, whereas Se application increased the abundance of *Fibrobacteres* in soil (**Figures 4A**, **6A**), thereby improving plant growth. The genus *Sphingomonas* has a strong metabolic capacity for aromatic compounds (Liu et al., 2019). The genus *Nitrosospira* plays a role in biological nitrification in the rhizosphere soil (Lin et al., 2018). Application of Se in Cd-contaminated soil increased the relative abundances of *Sphingomonas* and *Nitrosospira* in the rhizosphere soil, thus it improved the soil nitrogen cycle.

## 5 Conclusion

In this study, our results revealed that foliar spraying with 5 μM Se and the root application of 0.6 mg/kg or 1.2mg/kg Se improved Cd tolerance by decreasing Cd accumulation and increasing photosynthesis efficiency in perilla seedlings. The regulation of photosynthesis by Se is related to the promotion of chlorophyll synthesis and stability, maintaining normal stomata opening and increasing the photosynthetic electron transfer activity of the PS II reaction center. Root application of 1.2 mg/kg Se increased the contents of HC_1_ and HC_2_ in the cell wall under Cd stress. Moreover, root application of Se increased the relative abundance of *Proteobacteria*, *Bacteroides*, *Fibrobacteres*, *Sphingomonas* and *Nitrosspirillum* in Cd-contaminated soil, and reduced the relative abundance of *Actinomycetes*. Therefore, in Cd-contaminated soil, cultivating perilla with Se supplementation may promote the colonization of potentially beneficial bacteria, thereby altering the composition of the soil microbial community, finally affecting plant growth and Cd tolerance. These results provide a greater understanding of soil microbiome resiliency and the impacts of Cd as pollutant on the soil microbial communities.

## Data availability statement

The raw data supporting the conclusions of this article will be made available by the authors, without undue reservation.

## Author contributions

YL and JM designed and supervised the research. XY, YL, JM, and FW performed most experiments. XY, YL, JM, LW, LS, and PZ analyzed characterized the phenotypes. XY, YL, JM, and FW analyzed the data and WW, XY, YL, JM, JX, and FW wrote the manuscript. All authors contributed to the article and approved the submitted version.

## Funding

This research was supported by the Science and technology Innovation Fund project of Shanxi Agricultural University (2020BQ24 and 2020QC13) and the Basic Research Program of Shanxi Province (Free Exploration) (20210302124369 and 20210302124065).

## Conflict of interest

The authors declare that the research was conducted in the absence of any commercial or financial relationships that could be construed as a potential conflict of interest.

## Publisher’s note

All claims expressed in this article are solely those of the authors and do not necessarily represent those of their affiliated organizations, or those of the publisher, the editors and the reviewers. Any product that may be evaluated in this article, or claim that may be made by its manufacturer, is not guaranteed or endorsed by the publisher.
