# Supplementary material for: Comparative physiological and soil microbial community structural analysis revealed that selenium alleviates cadmium stress in Perilla frutescens
Source: Front Plant Sci. 2022 Oct 7;13:1022935. doi: 10.3389/fpls.2022.1022935 (PMC9585217; doi:10.3389/fpls.2022.1022935)
Supplement: Supplementary file 1 [file DataSheet_1.docx]

**Supplemental Tables and Figures**

**Table S1** Effects of foliar spraying selenium (5 µM Se) on seedling biomass of perilla under cadmium stress (10 mg/kg Cd). Data in the table are mean ± standard error.

| Treatments | Leaf fresh weight (g) | Leaf dry weight (g) | Root fresh weight (g) | Root dry weight (g) | Root length (cm) |
| --- | --- | --- | --- | --- | --- |
| control | 20.86±1.39a | 2.22±0.15a | 7.22±0.57a | 0.73±0.07a | 33.00±1.58b |
| Se | 20.24±0.95a | 2.19±0.13a | 7.14±1.14a | 0.66±0.05a | 38.58±0.55a |
| Cd | 19.19±1.96a | 1.82±0.24a | 5.94±0.78a | 0.52±0.06a | 29.65±0.31b |
| Se + Cd | 19.13±2.08a | 2.06±0.22a | 8.21±1.42a | 0.74±0.11a | 29.83±1.94b |

**Table S2** Effects of root exposure of selenium on seedling biomass of perilla under cadmium stress (10 mg/kg Cd). Data in the table are mean ± standard error.

| Treatments | Leaf fresh weight (g) | Leaf dry weight (g) | Root fresh weight (g) | Root dry weight (g) | Root length (cm) |
| --- | --- | --- | --- | --- | --- |
| control | 19.94±1.24a | 1.90±0.13a | 7.52±0.72a | 0.59±0.07a | 32.63±2.20a |
| 0.6 mg/kg Se | 20.07±2.03a | 1.95±0.18a | 7.76±1.08a | 0.62±0.10a | 30.75±3.29a |
| 1.2 mg/kg Se | 24.05±2.64a | 2.14±0.27a | 6.98±1.12a | 0.54±0.09a | 35.48±0.98a |
| Cd | 20.23±2.01a | 1.96±0.22a | 7.52±0.96a | 0.58±0.06a | 36.90±2.37a |
| 0.6 mg/kg Se + Cd | 22.93±1.30a | 2.24±0.10a | 8.63±0.87a | 0.67±0.06a | 37.13±1.63a |
| 1.2 mg/kg Se + Cd | 20.69±0.95a | 2.48±0.10a | 9.01±0.78a | 0.79±0.07a | 32.73±2.07a |

**Table S3** Statistical table of effective sequencing data of rhizosphere soil bacteria. Sample: Sequenced Sample name; RS: rhizosphere soil; Cd: 10 mg/kg Cd (soil application of exogenous cadmium); Se: 1.2 mg/kg Se (soil application of exogenous selenium); Raw_Tags: Sequenced original data, counting the number of sequencing sequences of each file in a unit of four reads; Raw_Bases: The number of sequences multiplied by the length of the sequence, expressed in unit M; Valid_Tags: After preprocessing, count the number of sequenced sequences after stitching of each file in a unit of four behaviors; Valid_Bases: After pretreatment, the number of sequencing sequences is multiplied by the length of the sequence, and is expressed in unit M; Valid%: Ratio of Valid data to Raw data, expressed as percentage; Q20%: the proportion of data with data quality ≥Q20 in valid data; Q30%: the proportion of data with data quality ≥Q30 in valid data; GC%: Data GC content in valid data.

| Sample | Raw_Tags | Raw_Bases | Valid_Tags | Valid_Bases | Valid% | Q20% | Q30% | GC% |
| --- | --- | --- | --- | --- | --- | --- | --- | --- |
| control _RS1 | 68354 | 28.28M | 68354 | 28.28M | 100.00 | 97.68 | 93.02 | 56.65 |
| control _RS2 | 62690 | 25.93M | 62690 | 25.93M | 100.00 | 97.44 | 92.30 | 57.06 |
| control _RS3 | 55585 | 22.94M | 55585 | 22.94M | 100.00 | 96.40 | 89.80 | 56.86 |
| control _RS4 | 58907 | 24.38M | 58907 | 24.38M | 100.00 | 97.69 | 93.06 | 56.71 |
| Se_RS1 | 68261 | 28.23M | 68261 | 28.23M | 100.00 | 97.60 | 92.84 | 56.70 |
| Se_RS2 | 57692 | 23.77M | 57692 | 23.77M | 100.00 | 97.01 | 91.54 | 56.79 |
| Se_RS3 | 67314 | 27.84M | 67314 | 27.84M | 100.00 | 96.73 | 90.49 | 56.63 |
| Se_RS4 | 63632 | 26.26M | 63632 | 26.26M | 100.00 | 96.69 | 90.73 | 56.64 |
| Cd_RS1 | 63317 | 26.15M | 63317 | 26.15M | 100.00 | 97.78 | 93.27 | 57.28 |
| Cd_RS2 | 58948 | 24.33M | 58948 | 24.33M | 100.00 | 97.86 | 93.52 | 57.08 |
| Cd_RS3 | 70730 | 29.21M | 70730 | 29.21M | 100.00 | 96.77 | 90.85 | 56.41 |
| Cd_RS4 | 60628 | 24.93M | 60628 | 24.93M | 100.00 | 97.99 | 93.76 | 57.77 |
| Cd Se_RS1 | 67072 | 27.73M | 67072 | 27.73M | 100.00 | 97.81 | 93.36 | 56.58 |
| Cd Se_RS2 | 61558 | 25.45M | 61558 | 25.45M | 100.00 | 97.73 | 93.11 | 56.93 |
| Cd Se_RS3 | 56817 | 23.48M | 56817 | 23.48M | 100.00 | 97.23 | 91.90 | 56.92 |
| Cd Se_RS4 | 62884 | 26.00M | 62884 | 26.00M | 100.00 | 97.65 | 92.92 | 57.10 |

**Table S4** Alpha Indices statistical table of bacteria in rhizosphere soil sample group. RS: rhizosphere soil; Cd: 10 mg/kg Cd (soil application of exogenous cadmium); Se: 1.2 mg/kg Se (soil application of exogenous selenium). Data in the table are mean ± standard error.

| Treaments | Observed_species | Shannon | Simpson | Chao1 | Goods _coverage |
| --- | --- | --- | --- | --- | --- |
| control _RS | 2123.75±220.73a | 9.84±0.17a | 1.00±0.00a | 2162.23 ±233.62a | 1.00±0.00a |
| Cd_RS | 2212.25±86.97a | 9.90±0.06a | 1.00±0.00a | 2268.06±89.27a | 0.99±0.00a |
| Se_RS | 2135.25±87.63a | 9.95±0.04a | 1.00±0.00a | 2179.24±102.36a | 1.00±0.00a |
| Cd Se_RS | 2247.25±109.21a | 9.95±0.08a | 1.00±0.00a | 2295.83±123.18a | 0.99±0.00a |

**Table S5** Statistical table of effective sequencing data of non-rhizosphere soil bacteria. Cd: 10 mg/kg Cd (soil application of exogenous cadmium); Se: 1.2 mg/kg Se (soil application of exogenous selenium); S: non-rhizosphere soil. Raw_Tags: Sequenced original data, counting the number of sequencing sequences of each file in a unit of four reads; Raw_Bases: The number of sequences multiplied by the length of the sequence, expressed in unit M; Valid_Tags: After preprocessing, count the number of sequenced sequences after stitching of each file in a unit of four behaviors; Valid_Bases: After pretreatment, the number of sequencing sequences is multiplied by the length of the sequence, and is expressed in unit M; Valid%: Ratio of Valid data to Raw data, expressed as percentage; Q20%: the proportion of data with data quality ≥Q20 in valid data; Q30%: the proportion of data with data quality ≥Q30 in valid data; GC%: Data GC content in valid data.

| Sample | Raw_Tags | Raw_Bases | Valid_Tags | Valid_Bases | Valid% | Q20% | Q30% | GC% |
| --- | --- | --- | --- | --- | --- | --- | --- | --- |
| control_S2 | 70431 | 28.83M | 70431 | 28.83M | 100.00 | 95.27 | 87.11 | 56.49 |
| control _S3 | 60579 | 25.06M | 60579 | 25.06M | 100.00 | 97.78 | 93.26 | 56.89 |
| control _S4 | 60429 | 24.93M | 60429 | 24.93M | 100.00 | 97.01 | 91.29 | 56.94 |
| Se_S1 | 61422 | 25.35M | 61422 | 25.35M | 100.00 | 97.73 | 93.07 | 56.60 |
| Se_S2 | 62049 | 25.57M | 62049 | 25.57M | 100.00 | 97.70 | 93.06 | 56.68 |
| Se_S4 | 60148 | 24.83M | 60148 | 24.83M | 100.00 | 96.31 | 89.63 | 56.45 |
| Cd_S1 | 64354 | 26.53M | 64354 | 26.53M | 100.00 | 95.85 | 88.79 | 56.63 |
| Cd_S2 | 66846 | 27.56M | 66846 | 27.56M | 100.00 | 96.02 | 88.71 | 56.50 |
| Cd_S3 | 67526 | 27.87M | 67526 | 27.87M | 100.00 | 97.71 | 93.08 | 56.73 |
| Cd_S4 | 66039 | 27.22M | 66039 | 27.22M | 100.00 | 96.51 | 90.04 | 56.66 |
| Cd Se_S1 | 66783 | 27.55M | 66783 | 27.55M | 100.00 | 96.85 | 90.89 | 56.61 |
| Cd Se_S3 | 62138 | 25.58M | 62138 | 25.58M | 100.00 | 97.81 | 93.34 | 56.77 |
| Cd Se_S4 | 74866 | 30.88M | 74866 | 30.88M | 100.00 | 96.14 | 89.16 | 56.71 |

**Table S6** Alpha Indices statistical table of bacteria in non-rhizosphere soil sample group. Cd: 10 mg/kg Cd (soil application of exogenous cadmium); Se: 1.2 mg/kg Se (soil application of exogenous selenium); S: non-rhizosphere soil. Data in the table are mean ± standard error.

| Treatments | Observed_species | Shannon | Simpson | Chao1 | Goods _coverage |
| --- | --- | --- | --- | --- | --- |
| control_S | 1858.67±374.79a | 9.61±0.28a | 1.00±0.00a | 1862.90 ±377.62a | 1.00±0.00a |
| Cd_S | 1657.00±177.77a | 9.47±0.13a | 1.00±0.00a | 1668.74±187.32a | 1.00±0.00a |
| Se_S | 1792.67±179.9a | 9.46±0.10a | 1.00±0.00a | 1801.08±184.10a | 1.00±0.00a |
| Cd Se_S | 1856.67±131.93a | 9.64±0.11a | 1.00±0.00a | 1867.63±131.86a | 1.00±0.00a |

**Supplemental figures**


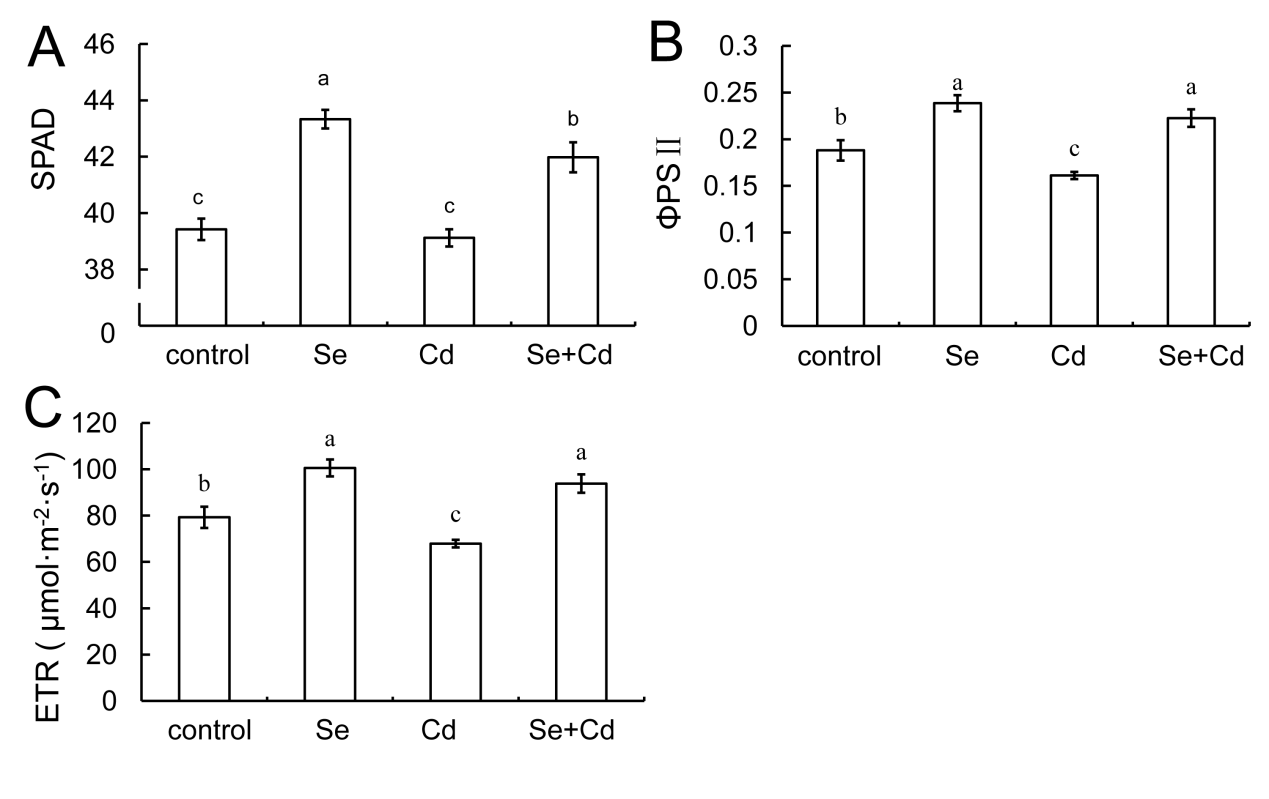


**Figure S1** Effects of foliar spraying Se (5 µM) on photosynthetic efficiency of perilla seedlings under Cd stress (10 mg/kg). A: SPAD; B: actual photochemical efficiency of PSII reaction centers; C: apparent photosynthetic electron transfer efficiency.


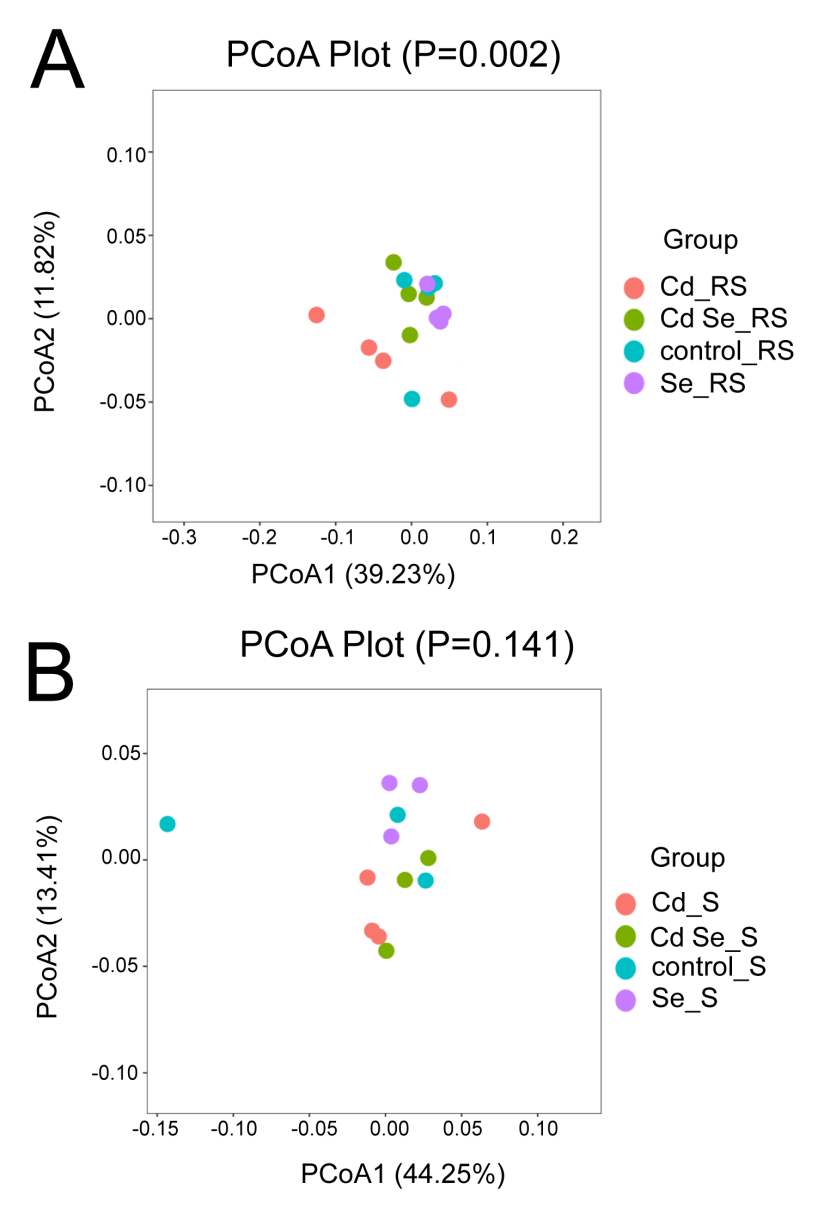


**Figure S2** Principal coordinate analysis diagram. A: rhizosphere soil; B: non-rhizosphere soil.


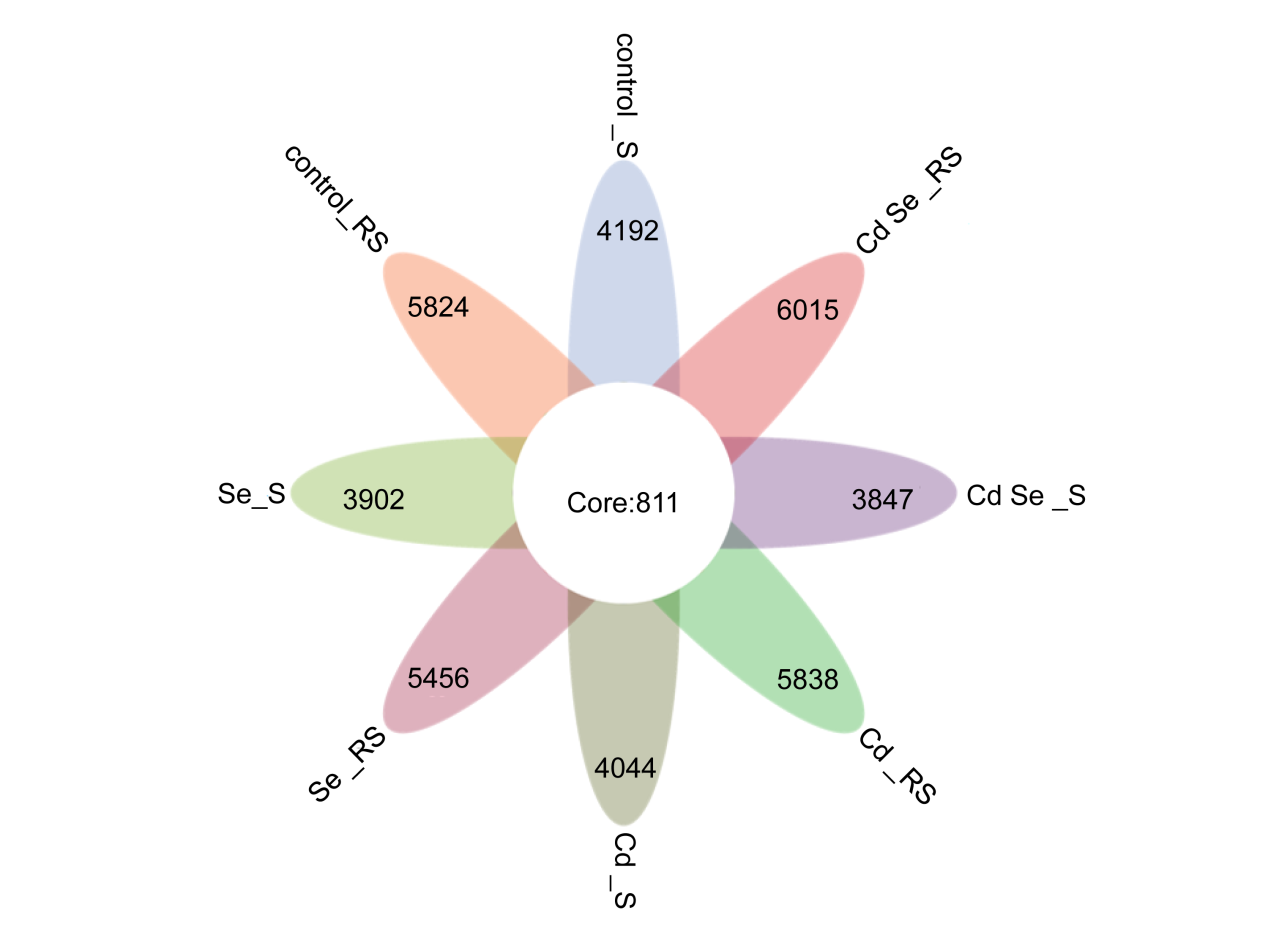


**Figure S3** Petal map of soil feature distribution.
